# Correlation between 25-hydroxyvitamin D level of lactating mothers and bone mineral density of infants and analysis of risk factors

**DOI:** 10.5937/jomb0-48471

**Published:** 2024-11-16

**Authors:** Yan Jin, Minghui Li, Wei Ding, Huiwen Wu

**Affiliations:** 1 Maternal and Child Health Hospital of Hubei Province, Department of Child Health, Wuhan, Hubei Province, China

**Keywords:** infants, bone mineral density, serum 25hydroxyvitamin D (25-(OH)D), sunshine duration, influencing factors, novorođenčad, mineralna gustina kostiju, serumski 25-hidroksivitamin D (25-(OH)D), trajanje sunčeve svetlosti, faktori uticaja

## Abstract

**Background:**

Aim was to demonstrate the influencing factors of infant bone mineral density (BMD) and its correlation with serum 25-hydroxyvitamin D (25-(OH)D) in nursing mothers.

**Methods:**

200 children aged 0 č 1 years were rolled into normal group (n=120) and abnormal group (n=80) regarding the results of ultrasound BMD examination. The sunshine duration of infants with different BMD and 25(OH)D, calcium and phosphorus levels of nursing mothers were analyzed, and univariate and multivariate analyses of BMD were implemented.

**Results:**

The results revealed that the sunshine duration and serum 25-(OH)D level of nursing mothers in abnormal group were inferior to those in normal group (P<0.05). Additionally, a notable positive correlation existed between sunshine duration, serum 25-(OH)D level of nursing mothers and BMD (r = 0.911 and 0.503, P<0.05). According to Logistic regression analysis, outdoor activity time 0 č 1 h/d, premature infants, and breastfeeding alone were independent risk factors (RFs) for abnormal BMD in infants, and vitamin D(VD) and calcium supplementation were independent protective factors (P<0.05).

**Conclusions:**

VD and calcium intake, adequate sunshine duration, mixed feeding, and increasing serum 25-(OH)D can reduce the occurrence of abnormal BMD in infants.

## Abstract (sr)

**Uvod:**

Cilj je bio da se demonstriraju faktori uticaja mineralne gustine kostiju novorođenčadi (BMD) i njihova korelacija sa serumskim 25-hidroksivitaminom D (25-(OH)D) kod dojilja.

**Metode:**

200 dece uzrasta od 0 do 1 godine uvršteno je u normalnu grupu (n=120) i abnormalnu grupu (n=80) prema rezultatima ultrazvučnog pregleda BMD. Analizirano je trajanje sunčeve svetlosti odojčadi sa različitim BMD i 25-(OH)D, kalcijuma i fosfora dojilja i primenjene su univarijantne i multivarijantne analize BMD.

**Rezultati:**

Rezultati su pokazali da su trajanje sunčeve svetlosti i nivo 25-(OH)D u serumu dojilja u abnormalnoj grupi inferiorni u odnosu na one u normalnoj grupi (P<0,05). Pored toga, postojala je primetna pozitivna korelacija između trajanja sunčeve svetlosti, nivoa 25-(OH)D u serumu kod dojilja i BMD (r = 0,911 i 0,503, P<0,05). Prema logističkoj regresionoj analizi, vreme aktivnosti na otvorenom 0 č 1 h/d, prevremeno rođena beba i samo dojenje bili su nezavisni faktori rizika (RF) za abnormalnu BMD kod novorođenčadi, a vitamin D(VD) i suplementacija kalcijuma su bili nezavisni zaštitni faktori (P <0,05).

**Zaključak:**

Unos VD i kalcijuma, adekvatno trajanje sunčeve svetlosti, mešano hranjenje i povećanje serumskog 25-(OH)D mogu smanjiti pojavu abnormalne BMD kod odojčadi.

## Introduction

Infants and children are at the first peak of body development, during which bone mass increases rapidly and accumulates. Osteopenia is an important factor in reducing the occurrence of rickets in childhood and osteoporosis in adulthood [1]. Bone mineral density (BMD) is the content of mineral levels in tissues and organs and can assess bone nutritional status [2]. Insufficient BMD has been demonstrated to lead to the development of fractures and osteoporosis [3] [4]. The abnormal rate of BMD in infants has been increasing, while the fracture rate and rickets rate in infants with BMD deficiency are drastically superior to those in infants with normal BMD [5]. Periodic monitoring of BMD in infants and young children and guiding the administration of vitamin D (VD) or calcium supplements are imperative for the prevention of diseases caused by BMD deficiency.

VD is a micronutrient, which is involved in calcium and phosphorus metabolism and bone development and is crucial for preventing the occurrence of rickets in children [6]. VD can promote the body to absorb calcium, and mobilize the calcium components in bone, and then maintain the calcium level in plasma, and ultimately promote the normal development and growth of bone and structural reconstruction in infants [7]. VD deficiency in pregnant women leads to an enhanced risk of low birth weight and premature infants [8]. It has also been confirmed that VD deficiency during pregnancy increases the incidence of gestational diabetes and preeclampsia [9] [10]. 25-hydroxyVD (25-(OH)D) belongs to endogenous and exogenous VD, which has the advantages of long half-life, high concentration, and relative stability *in vivo* and is an imperative metabolite of VD [11]. 25-(OH)D can be used to assess VD nutrition in humans. Maternal nutrients can deliver nutrients to the fetus through the umbilical cord, and maternal calcium nutrition can directly affect the bone growth of fetus and newborn [12].

However, there are relatively few studies on correlation between maternal 25-(OH)D and BMD in infants. It aimed to investigate the correlation between ultrasound BMD of infants aged 0 ~ 1 years and serum 25-(OH)Din nursing mothers, and to analyze risk factors (RFs) affecting BMD in infants. The aim is to understand the relationship between BMD and VD levels in nursing mothers, and to provide reference materials for the importance of VD and calcium during pregnancy.

## Materials and methods

### General data

A total of 200 children aged 0~1 years who visited Maternal and Child Health Hospital of Hubei Province from December 2021 to March 2023 and received BMD examination were recruited and rolled into normal group (n = 120) and abnormal group (n = 80) based on the BMD examination outcomes. Inclusion criteria: children receiving bone densitometer examination at admission; children aged 0 ~ 1 years old; children without hereditary bone metabolism disease, or other diseases that can affect the level of bone metabolism. Exclusion criteria: children with liver or kidney dysfunction; children with endocrine metabolism or immune dysfunction; children with osteitis deformans, osteogenesis imperfecta, and various bone metabolic diseases; children with respiratory system or digestive system diseases; pregnant women who have been using hormone drugs for a long time during pregnancy; children whose mothers have severe metabolic diseases during lactation; children with a history of fracture or traumatic diseases. The family members of the children signed the informed consent form, and the trial conformed to the approval of the medical ethics committee of the hospital (HWH2021-L01).

### Bone density testing

XR-600 digital fast dual-energy X-ray densitometer produced by Norland was used to scan BMD in the left middle tibia of the child. Based on the relevant standards set by WHO, BMD is defined as abnormal if it is less than 2.5 standard deviations from the mean peak bone mass of healthy people of the same sex, or a decrease of more than 30%. When T value>-1.0, bone density was considered normal. When T value is in the range of -2.5 ~ -1.0, bone mass decreases. When T value ≤ -2.5, it is osteoporosis [13].

### Detection of serum 25-(OH)D, calcium, and phosphorus

5 mL of fasting blood was collected from nursing mothers, and serum was collected for 25-(OH)D, calcium, and phosphorus levels after anticoagulant treatment. Serum 25-(OH)D was determined employing the 25-(OH)D assay kit (Shanghai mlbio) under a Beckman Coulter UniCel Dxl 800 automated chemiluminescent immunoassay system [14]. Serum calcium and phosphorus levels were determined using an electrolyte test kit (Shanghai Tellgen Corporation) on a Beckman Coulter AU5821 automatic biochemical analyzer.

### Statistical analysis

Using SPSS 19.0, enumeration data were denoted as n (%) and tested by χ^2^ test. Measurement data were denoted as mean ± SD, and *t*-test was performed. Spearman’s test was adopted for correlation analysis of 25-(OH)D in nursing mothers with infant BMD. Influencing factors of infant BMD were analyzed using multivariate logistic regression. Differences were statistically significant if *P*<0.05.

## Results

### Demographic data of included population

A total of 200 infants including 120 in normal and 80 in abnormal group were included. A total of 103 infants were male and 97 were females. Ninety-nine infants were fed with breast milk while 28 using artificial milk and 73 were fed using mixed methods.

### Differences in 25-(OH)D in nursing mothers of infants with different BMD

**Figure 1:**
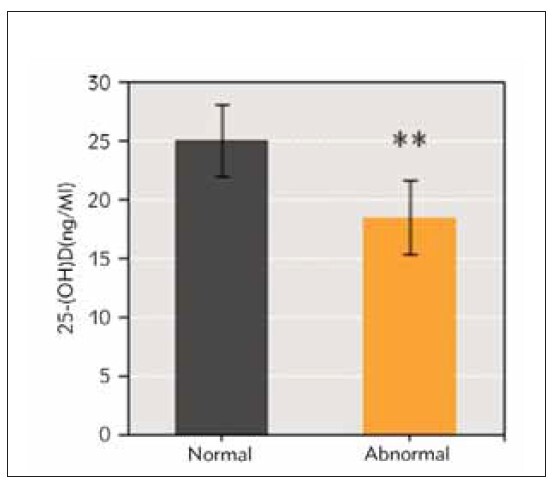
Contrast of 25-(OH)D in nursing mothers of infants with normal and abnormal BMD. (**P<0.01 vs. normal BMD group)

Serum 25-(OH)D level of nursing mothers in normal group and abnormal group was (25.13 ± 3.09) ng/mL and (18.59 ± 3.14) ng/mL. That of nursing mothers in abnormal group was markedly inferior to normal group (*P*<0.01) (Figure 1).

### Differences in calcium and phosphorus levels in nursing mothers of infants with different BMD

**Figure 2:**
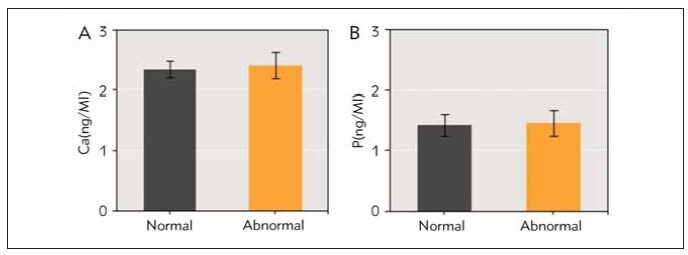
Comparison of serum calcium and phosphorus levels in nursing mothers of infants with normal and abnormal BMD. (A is comparison of serum calcium levels; B is comparison of serum phosphorus levels)

Serum calcium level was (2.35 ± 0.14) ng/mL in normal group and (2.41 ± 0.22) ng/mL in abnormal group; serum phosphorus level was (1.43 ± 0.18) ng/mL in normal group and (1.46 ± 0.20) ng/mL in abnormal group. Neglectable difference was found in serum calcium and phosphorus levels between two groups (*P*>0.05) (Figure 2).

### Differences in sunshine duration in infants with different BMD

**Figure 3:**
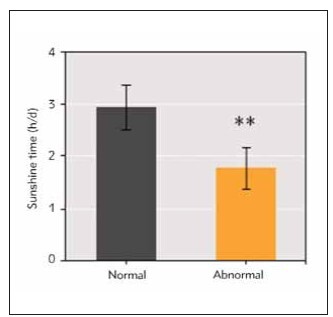
Comparison of sunshine duration between infants with normal BMD and infants with abnormal BMD. (**P<0.01 vs. normal BMD group)

The duration of sunshine was (2.95 ± 0.43) h/d in normal group and (1.79 ± 0.39) h/d in abnormal group. The sunshine duration of abnormal BMD group was markedly inferior to that of normal BMD group (*P*<0.01) (Figure 3).

### Correlation between BMD and 25-(OH)D and sunshine duration

**Figure 4:**
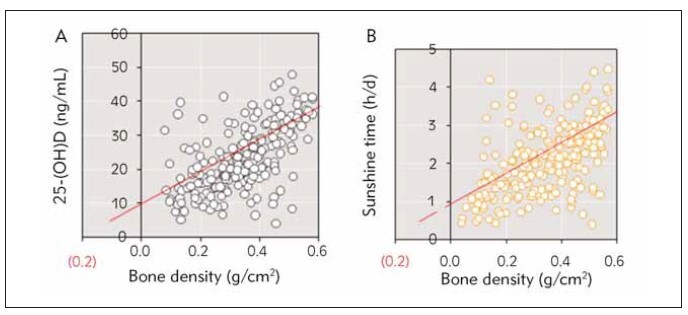
The correlation between BMDP value and 25-(OH)D level in nursing mothers and sunshine time. (A is the correlation between BMD and 25-(OH)D in nursing mothers; B is the correlation between BMD and sunshine time)

The correlation between BMD*P* value and 25-(OH)D in nursing mothers and sunshine duration was analyzed. The results showed a notable positive correlation between serum 25-(OH)D level and BMD (*r*=0.911, *P*<0.01) and between sunshine duration and BMD (*r*=0.503, *P*<0.01) (Figure 4).

### Univariate analysis affecting infant BMD

Univariate analysis affecting infant BMD was performed. It was found that no considerable difference existed in gender between children with normal BMD and abnormal BMD (*P*>0.05). It was found that the proportion of breast feeding, outdoor exercise time 0 ~ 1 h, and premature infants with abnormal BMD was drastically superior to that of infants with normal BMD (*P*<0.05), while the proportion of VD supplementation and calcium supplementation of infants with abnormal BMD was markedly inferior to that of infants with normal BMD (*P*<0.05) (Table 1).

**Table 1:** Univariate analysis of infant BMD.

Factors		Normal BMD <br>(*n*=120)	Abnormal BMD <br>(*n*=80)	*χ^2^*	*P*
Gender	Male	64 (53.3)	39 (48.8)	0.035	0.637
	Female	56 (46.7)	42 (52.5)		
Feeding methods	Breast milk	49 (40.8)	50 (62.5)	5.231	0.023
	Artificial	20 (16.7)	8 (10.0)		
	Mixed	51 (42.5)	22 (27.5)		
VD supplementation	Yes	82 (68.3)	41 (51.3)	10.829	0.001
	No	38 (31.7)	39 (48.8)		
Calcium supplementation	Yes	73 (60.8)	35 (43.8)	4.306	0.029
	No	47 (39.2)	45 (56.3)		
Outdoor sports	0 ~ 1 h	33 (27.5)	34 (42.5)	7.450	0.015
	1 ~ 2 h	41 (34.2)	25 (31.3)		
	More than 2 h	46 (38.3)	21 (26.3)		
Premature infants	Yes	11 (9.2)	26 (32.5)	11.438	0.001
	No	109 (90.8)	54 (67.5)		

### Multivariate analysis of factors affecting BMD in infants

It was found that breast feeding, outdoor exercise time less than 1 h/d, and premature infants were independent RFs for abnormal BMD (*P*<0.05). VD supplementation and calcium supplementation were independent protective factors for normal BMD in infants (*P*<0.05) (Table 2).

**Table 2:** Logistic multivariate regression analysis of infant BMD.

Factors	*β*	*S.E.*	*Wald χ^2^*	*P*	*OR*	*95% CI*	
						Lower limit	Upper limit
Breastfeeding alone	0.611	0.202	4.132	0.023	1.522	1.108	1.953
VD supplementation	-0.782	0.230	3.809	0.018	0.738	0.259	1.316
Calcium<br>supplementation	-0.533	0.159	3.981	0.036	0.446	0.180	0.839
Outdoor sports	-0.575	0.281	4.056	0.007	2.035	1.633	2.652
Premature infants	0.894	0.154	4.228	0.019	3.129	1.748	5.609

## Discussion

Infants aged 0 ~ 3 years are in an important and critical period of bone growth and development, insufficient exercise, malnutrition, and abnormal BMD can affect the growth and development of infants, and in severe cases, it can also lead to osteomalacia, fracture, and rickets [15] [16]. BMD can directly reflect the body calcium nutrition level, and regular monitoring of BMD can effectively assess the bone health status of infants [17]. The BMD of infants aged 0 ~ 3 years was evaluated by ultrasound BMD, and the correlation between BMD and 25-(OH)D level in nursing mothers was analyzed according to the measured *P* value as the evaluation criteria of bone strength in infants. VD is an important steroid hormone, which can promote the absorption of calcium and phosphorus levels in the body, is very important for maintaining stable calcium levels and bone health, and can also regulate the body’s cellular immune function [18]. 25-(OH)D is a combined form of endogenous and exogenous VD, which is the main degree of VD metabolism and can be used for the evaluation of VD levels in the body [19]. The results showed that 25-(OH)D in nursing mothers of infants with abnormal BMD was markedly inferior to those in nursing mothers of infants with normal BMD. After pregnancy and childbirth, nursing mothers lose large amounts of nutrients, including VD. Breast milk contains very little VD, so breastfeeding alone makes it impossible for infants to obtain enough VD from breast milk [20]. It is consistent with the finding that breastfeeding alone is an independent RF for abnormal BMD in infants and young children.

It has been confirmed that VD in nursing mothers is influenced by factors such as age, ethnicity, sunshine duration, and calcium supplementation [21] [22] [23]. VD is a lipid-soluble steroid derivative, and 90% of VD is derived from skin production after UV irradiation [24]. The differences of sunshine duration in infants with different BMD were compared, and it was found that the sunshine duration in infants with abnormal BMD was greatly shorter than that in infants with normal BMD. Outdoor exercise with adequate sunshine duration can greatly improve the absorption of calcium and phosphorus by VD in the body, increasing BMD [25] [26]. It is consistent with the finding of a positive correlation between sunshine duration and ultrasound BMD. The RFs affecting BMD in infants were further analyzed, it was found that outdoor activity time 0 ~ 1 h/d, premature infants, and breastfeeding alone were independent RFs for BMD abnormalities in infants. The younger the age, the less the mineral level deposition in the bone, the immature bone development, while insufficient activity, irregular diet will cause a decrease in BMD [27]. Shorter outdoor time reduces the duration of UV exposure to the skin of infants, reduces active VD levels, and then reduces the body’s absorption of calcium and phosphorus levels, leading to BMD deficiency [28]. Premature infants are prone to bone deficiency because of reduced reserves of iron, vitamin A, and VD, and shortened intrauterine activity, which is not conducive to bone growth and development as well as bone mass accumulation [29].

In addition, VD supplementation and calcium supplementation were found to be independent protective factors for abnormal BMD in infants. Maternal nutritional status has an important impact on infants and young children, and the main sources of maternal VD supplementation are dietary intake and sunshine, of which dietary intake of VD content is very small. It has been confirmed that exogenous VD supplementation during lactation can greatly increase VD levels in nursing mothers [30]. Calcium and other minerals during pregnancy are mainly obtained through factors such as diet and intestinal secretion and reabsorption, and adequate calcium intake during pregnancy can make infants have normal bone mass and maintain normal BMD levels after birth [31]. Postpartum calcium supplementation can participate in bone metabolism, and assist bone formation and deposition, improving the BMD level of infants. In summary, it is recommended to pay attention to VD and calcium supplementation during pregnancy and postpartum period, advocate breast milk combined with milk powder mixed feeding, increase the outdoor activities of adequate sunshine for pregnant women and postpartum infants, and encourage infants to receive regular ultrasound BMD examination to reduce the risk of a series of diseases caused by BMD deficiency in infants.

## Conclusion

25-(OH)D and sunshine duration were greatly positively correlated with BMD in nursing mothers. Among infants aged 0 ~ 1 years, outdoor activity time 0 ~ 1 h/d, premature infants, and breastfeeding alone were independent RFs of abnormal BMD in infants, while VD and calcium supplementation were independent protective factors. The relationship between 25-(OH)D in nursing mothers and BMD in infants was analyzed only. The changes of 25-(OH)D in infants were not detected, and the intake of VD and calcium in nursing mothers was not analyzed. Additional information needs to be included to supplement these gaps in the future. The results can provide scientific guidance for VD and calcium intake in perinatal period, and prevent the occurrence of fracture, osteomalacia, and rickets in infants caused by abnormal BMD.

## Dodatak

### Conflict of interest statement

All the authors declare that they have no conflict of interest in this work.
